# Comparing Metabolic Preconditioning and Diabetes As Risk Factors in Knee Arthroplasty Complications

**DOI:** 10.7759/cureus.56634

**Published:** 2024-03-21

**Authors:** Gelu F Murvai, Timea Claudia Ghitea, Simona Cavalu

**Affiliations:** 1 Surgery Department, Faculty of Medicine and Pharmacy, University of Oradea, Oradea, ROU; 2 Pharmacy Department, University of Oradea, Oradea, ROU; 3 Therapeutics Department, Faculty of Medicine and Pharmacy, University of Oradea, Oradea, ROU

**Keywords:** nsaids, knee injuries, osteoarthritis, metabolic preconditions, diabetes

## Abstract

Background and objectives: Advanced osteoarthritis of the knee joint severely affects the patient's mobility, compounded by pre-existing comorbidities such as metabolic preconditioning (such as obesity, dyslipidemia, hyperuricemia, and insulin resistance syndrome) and both type I and type II diabetes. The success of total knee arthroplasty is influenced by knowledge and management of risk factors. The present study aims to evaluate differences in the evolution of risk factors such as obesity, injuries, and sedentary lifestyle, distinguishing those with metabolic preconditions and diabetes. The objectives of our study include (1) investigating the prevalence of obesity among patients, highlighting their proportion in the five categories of body weight; (2) analyzing statistically significant differences between research groups in terms of weight status and physical activity; (3) evaluating postoperative evolution based on the administration of nonsteroidal anti-inflammatory drugs (NSAIDs) and without NSAIDs (N-NSAIDs), with an emphasis on overweight patients and those with diabetes; and (4) examining changes in metabolic preconditioning and the incidence of postoperative injury depending on the administration of anti-inflammatory drugs.

Materials and methods: A cohort involving 730 patients diagnosed with gonarthrosis was divided into two groups according to the administration of anti-inflammatory drugs in the first seven postoperative days: N-NSAIDs group (394 patients, 55.3%) and respectively NSAIDs group (319 patients, 44.7%). The prospective, observational study was conducted in terms of risk factors and complications that occurred upon treatment administration in relation to each type of intervention and implant used. The outcomes were assessed in terms of the influence on quality of life, the data being collected and interpreted for the entire cohort, and for each study year individually.

Results: The results indicate that almost 69% of them were overweight, while only 31% had a normal weight. Significant differences in weight status were observed between research groups, highlighting the association between obesity and metabolic preconditions or diabetes. Physical activity was absent in a significant proportion, having a notable impact on postoperative evolution, especially in the group without metabolic precondition. Administration of anti-inflammatory drugs influenced postoperative outcomes, with significant differences in overweight and diabetic patients.

Conclusions: The findings suggest the need to manage body weight, promote physical activity, and personalize postoperative treatments, given the complex interactions between obesity, metabolic preconditions, and the administration of NSAIDs.

## Introduction

Osteoarthritis was traditionally considered a degenerative joint disease characterized by progressive loss of articular cartilage. However, recent studies have emphasized its complexity, attributing its origins to multiple causes, including inflammation, metabolic disorders, trauma, biochemical reactions, and mechanical forces [1]. It is crucial to note that cartilage is not the sole affected component in osteoarthritis [1-3]. Pain and inflammation in the early stages of the disease primarily result from changes in other joint components including ligaments, joint capsule, periarticular muscles, and synovium [1-3]. As the disease progresses, these components undergo changes, leading to evident alterations like periarticular muscle weakening, bone remodeling, synovial effusion, ligament weakening, and osteophyte formation [4]. Presently, the precise contributions of mechanical and metabolic components to joint structural changes, as well as the significance of metabolic "initiating" factors compared to mechanical ones, remain unclear [5].

In the case of obese individuals, the pathogenesis of primary arthrosis may commence with the expansion of adipose tissue [6], followed by alterations in adjacent tissue, as a consequence of increased synthesis of leptins [7,8]. Modifications in cartilage structure and morphology may also occur as a result of inflammation in excessive adipose tissue [9]. Mechanical overload and metabolic components contribute to establishing a positive feedback loop, making it challenging to break this vicious cycle [10]. This study identified metabolic disorders as a significant risk factor, affecting 38.14% of patients. The significance of adipokine levels in obese individuals may be particularly noteworthy since obesity can create a biochemical environment influencing the response of chondrocytes to such stimuli.

For instance, chondrocytes from patients with obesity and osteoarthritis exhibit a different response pattern to leptin compared to normal weight or overweight patients, while senescent chondrocytes are characterized by increased pro-catabolic activity. Despite its systemic anti-inflammatory effects, the role of adiponectin in joint diseases remains poorly understood, with conflicting reports on its pro-inflammatory and anti-inflammatory properties. A study emphasized that the impact of associated diseases and post-surgical complications on the physical function of patients who underwent total hip replacement is more significant than the influence of BMI itself [11]. Recently, two classifications were considered based on several clinical trials, shedding light on rapidly progressive osteoarthritis [12-14].

Rapidly progressive osteoarthritis type 1 described by Lequesne [14] involves a reduction in joint space width of over 2 mm within a year. In contrast, rapidly progressive osteoarthritis type 2 is characterized by massive bone loss and severe injury such as osteolysis [12,13]. In clinical trials involving anti-nerve growth factor (NGF) treatments, these types of rapidly progressive osteoarthritis are generally observed (though not always) in joints with pre-existing osteoarthritis [15]. On the other hand, research focused on accelerated knee osteoarthritis (AKOA) has concentrated on disease development [16-18].

To establish the initial health status of the cohort following the anamnesis, the goal was to determine initially existing risk factors (obesity, trauma, sedentarism), depending on people with metabolic disorders and diabetes. The present study aims to evaluate differences in the evolution of risk factors such as obesity, injuries, and sedentary lifestyle, distinguishing those with metabolic preconditions and diabetes. The diabetic precondition criterion included obesity, dyslipidemia, hyperuricemia, and insulin resistance syndrome confirmed by the Homeostatic Model Assessment (HOMA) index >2.00 (insulinemia (mU/L) x blood glucose (mmol/L))/22.5). The objectives of our study include (1) investigating the prevalence of obesity among patients, specifically focusing on the distribution across the five categories of body weight; (2) analyzing statistically significant differences between research groups in terms of weight status and physical activity; (3) evaluating postoperative progress based on the administration of nonsteroidal anti-inflammatory drugs (NSAIDs) and without NSAIDs (N-NSAIDs), with an emphasis on overweight patients and those with diabetes; and (4) examining changes in metabolic preconditioning and the incidence of postoperative injury depending on the administration of anti-inflammatory drugs.

## Materials and methods

All patients underwent total knee arthroplasty (TKA), during which risk factors such as metabolic disorders and biochemical reactions were obtained from the patient's medical history. Also, the patients considered in this study had the same type of implant, with no differences in manufacturer or material. Parameters such as obesity, injuries in three months (including both post-operative and inflammatory trauma), physical activity such as specific physiotherapy for postoperative recovery in six weeks, and associated pathologies were tracked separately and then compared based on the presence or absence of metabolic preconditions and the presence or absence of diabetes (type I and type II). The patients were diagnosed with diabetes before surgery. Obesity, according to WHO standards, is defined as patients with a BMI greater than 24.9 kg/m². The verification of patients was consistent with this criterion. The cohort was divided into two groups depending on the type of allopathic treatment administered. Thus, the first group of N-NSAIDs (394 patients, 55.3%) did not receive NSAIDs; instead, they were only administered analgesics, anti-algesics, and antidepressants in the first seven postoperative days. The other group (NSAIDs, 319 patients, 44.7%) received COX2-specific NSAIDs (celecoxib, etoricoxib) as prescribed treatment. The selection of patients was randomized (random.org), but the surgeon also took into account pre-existing pathologies or possible complications. Participation in the study was voluntary, and the patients were consecutively recruited from the Department of Orthopedics and Traumatology, Emergency County Clinical Hospital in Oradea, Romania, during the period 2018-2023, the study being approved by the Institutional Review Board and Ethical Council (approval no. CEFMF/1 dated 29.11.2023). The prospective, observational study was conducted in terms of risk factors and complications that occurred upon treatment administration in relation to each type of intervention and implant used. The outcomes were assessed in terms of the influence on quality of life, the data being collected and interpreted for the entire cohort, and for each study year individually. The inclusion criteria were patients with AKOA with or without previous surgery, age>18, who agreed to sign the informed consent. The exclusion criteria were patients who refused to sign the informed consent agreement, the existence of severe associated diseases, including serious neuropsychiatrical problems that may influence the patient monitoring. Decompensated or uncontrolled diabetes, as well as the onset of diabetes, were excluded from the study, in order not to mask the results. From the initially selected groups, 17 patients refused to continue the study, and hence, they were excluded due to missing follow-up.

Clinical analysis

At different time points (two weeks, six weeks, three months, and respectively six months), post-operative assessment was performed, while clinical parameters (such as type of intervention, surgical approach, and complications) were categorized into risk factors of knee arthropathy development.

Statistical analysis

Statistical analysis was performed using the Statistical Package for the Social Sciences (IBM SPSS Statistics for Windows, IBM Corp., Version 21, Armonk, NY). The results were analyzed by a univariate analysis of variance (ANOVA), in order to assess statistical differences between study groups, while Chi-square was utilized for non-parametric statistical analysis along with Skewness and Kurtosis statistical tests and Bonferroni multiple comparison between paired samples. Statistical significance was set at p<0.05.

## Results

The study cohort, comprising 713 patients, was selected from those presenting themselves at the County Clinical Hospital Orthopedics Department during the period 2018-2022. The average age was 52.48±11.78, with 386 (54.1%) men and 327 (45.9%) women. Urban origin environment was reported by 297 people (41.7%), and rural origin by 416 (58.3%). Table 1 provides a demographic description of the study groups with insignificant statistical differences (p > 0.05) in risk factors (metabolic precondition and diabetes).

**Table 1:** Demographic description M=male, F=female, U=urban, R=rural, SD=standard deviation, N=number of patients, NSAIDs=anti-inflammatory treatment group, N-NSAIDs=without anti-inflammatory treatment group

Parameters		Metabolic precondition								Diabetes							
		No				Yes				No				Yes			
		N-NSAIDs		NSAIDs		N-NSAIDs		NSAIDs		N-NSAIDs		NSAIDs		N-NSAIDs		NSAIDs	
		N	%	N	%	N	%	N	%	N	%	N	%	N	%	N	%
Gender	M	166	70.9	80	38.6	82	51.2	58	51.8	145	64.2	70	40.2	103	61.3	68	46.9
	F	68	29.1	127	61.4	78	48.8	54	48.2	81	35.8	104	59.8	65	38.7	77	53.1
Environment	U	100	42.7	78	37.7	65	40.6	54	48.2	90	39.8	77	44.3	75	44.6	55	37.9
	R	134	57.3	129	62.3	95	59.4	58	51.8	136	60.2	97	55.7	93	55.4	90	62.1
Age (mean±SD)		48.42±12.63		58.03±7.54		46.82±12.16		58.78±7.76		48.52±12.55		57.91±7,30		46.77±12.28		58.75±7.97	

**Figure 1:**
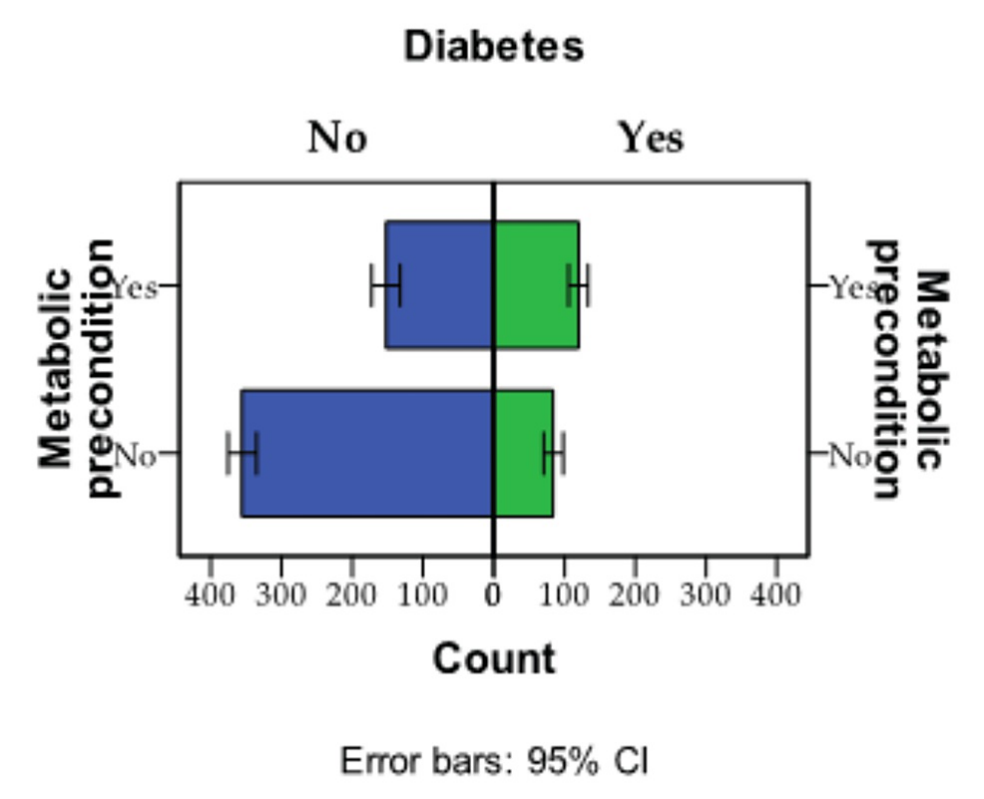
Graphic presentation of the two metabolic conditions - metabolic precondition and diabetes

The observed difference in significance (p=0.003) between metabolic precondition and diabetes was evident in the N-NSAIDs group (394 patients 55.3%), while it was deemed insignificant in the NSAIDs group (319 patients 44.7%), as illustrated in Figure 1. The cohort was tracked based on two criteria: metabolic precondition (272 people) and diabetes (313 people).

**Figure 2:**
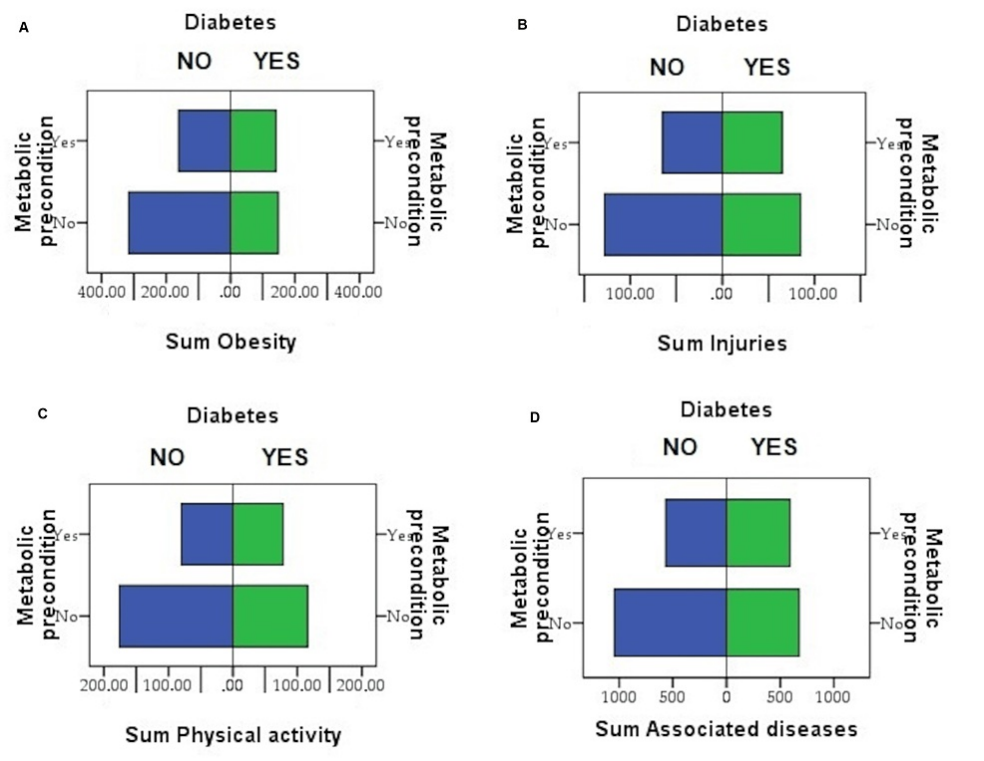
Histogram graphic representation of risk factors - obesity (A), Trauma (B), physical activity (C), associated diseases (D) - comparison between those with metabolic preconditions and those with diabetes.

Obesity stands out as one of the most significant risk factors for both osteoarthritis and fractures [19,20]. The weight status of patients was assessed by categorizing them into the five internationally recognized categories: normoponderal, overweight, obesity degree 1, obesity degree 2, and obesity degree 3. Notably, only 31.13% of the patients exhibited a body weight within the range of 18.5-24.99 kg/m^2^, corresponding to normal weight. Among the 68.87% classified as overweight, 44.03% had a BMI between 25 and 39.9 kg/m^2^, placing them in the overweight stage. A statistically significant difference in terms of weight status was identified between the research groups (p=0.017), specifically those with metabolic preconditions and diabetes (Figure 2). The complications of the prosthesis were followed in three months and six months, the results being published in other previous publications. It was observed a statistically insignificant relationship between postoperative complications such as fractures and diabetes, so this aspect was excluded. Table 2 shows the frequency and percentage distribution of the various postoperative complications investigated in the study. Variables include different types of postoperative complications. Thus, 57.8% of cases did not present postoperative complications. Surgical site infections occurred in 7.3% of cases, totaling up to 65.1% when combined with uncomplicated cases. Metabolic complications as delayed wound healing, dyslipidemia, and hyperglycemia, affected 14.4% of cases, contributing to a cumulative percentage of 79.5%. Significant differences were observed between the study groups regarding the administration of NSAIDs within the first seven days or without them, particularly in the occurrence of metabolic and respiratory complications. Additionally, a statistically significant difference was noted between those who did not experience any complications, with the majority being from the N-NSAIDs group. A 13.1% higher prevalence was recorded in individuals with a metabolic precondition for surgical site infection, with the difference being statistically significant. For other complications, increased prevalence was observed in the metabolic precondition group, but without significant differences.

**Table 2:** Investigated postoperative complications p=statistically significant, **Correlation is significant at the 0.01 level (2-tailed), *Correlation is significant at the 0.05 level (2-tailed).

Postoperative complications			Groups				p
Variables	Frequency	%	N-NSAIDs		NSAIDs		
			Frequency	%	Frequency	%	
Without complication	412	57.8	276	70.1	136	42.6	0.001**
Surgical site infection	52	7.3	23	5.8	29	9.1	0.405
Metabolic complication	103	14.4	63	16.0	40	12.5	0.023*
Periprosthetic joint infection	52	7.3	0	0.0	52	16.3	-
Respiratory complications	27	3.8	5	1.3	22	6.9	0.001**
Cardiovascular complications	39	5.5	15	3.8	24	7.5	0.150
Sepsis	28	3.9	12	3.0	16	5.0	0.450
Total	713						

Observations revealed that the issue of obesity extends beyond those who have already developed a metabolic disorder or diabetes. Surprisingly, a considerable proportion of individuals without metabolic preconditions and without diabetes also fell into the category of obesity. In this study was observed a positive, directly proportional, statistically significant correlation (p<0.05) between diabetes and metabolic precondition. Additionally, a statistically significantly higher number of patients with complications was recorded in the NSAIDs group.

Physical activity was notably absent in 63.11% of all patients, with statistically insignificant differences between groups (p>0.05) among those with a metabolic precondition and those with diabetes. However, a significant difference (p=0.001) was observed in those without a metabolic precondition and, respectively, without diabetes.

**Figure 3:**
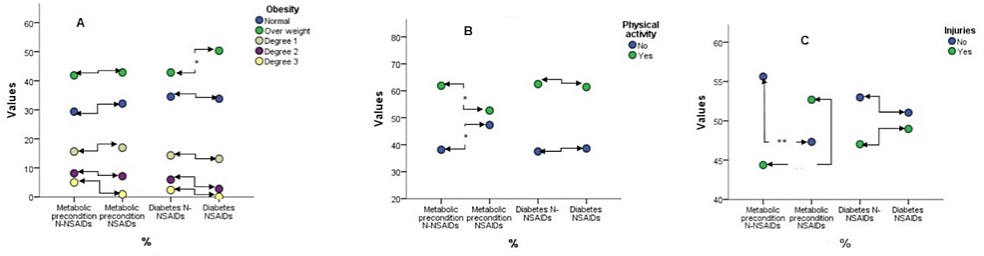
Graphic representation of differences in obesity (A), physical activity (B), and injuries (C) between those with metabolic preconditions and diabetes based on the N-NSAIDs and NSAIDs groups. NSAIDs=anti-inflammatory treatment group, N-NSAIDs= without anti-inflammatory treatment group

Regarding the differences in postoperative evolution between the N-NSAIDs and NSAID groups, significant disparities were noted, particularly in the case of overweight individuals with diabetes (Figure 3A). Noteworthy outcomes were also observed concerning physical activity in the metabolic precondition group, encompassing both those engaged in physical activity and those who are not (Figure 3B), as well as injuries (Figure 3C), concerning postoperative evolution with respect to N-NSAIDs and NSAIDs.

An increase in metabolic precondition is observable in individuals without physical activity using NSAIDs, whereas a decrease in metabolic precondition is noted in those engaging in physical activity and abstaining from anti-inflammatory drugs in the initial seven days postoperatively. Injuries exhibit a statistically significant decrease (p=0.001) in those using N-NSAIDs within the metabolic precondition group, while a significant increase (p=0.001) is observed in those using NSAIDs within the same metabolic precondition group.

Notably, no significant differences were observed in the case of diabetes, in terms of both physical activity and injuries. This highlights the heightened significance of metabolic preconditions in influencing postoperative outcomes.

## Discussion

When assessing the link between obesity and osteoarthritis risk, BMI has proven to be a valuable tool, despite ongoing debates about which obesity measure best correlates with this risk. According to research by Lohmander et al. (2009) [19], BMI was found to be strongly correlated with severe osteoarthritis rather than other anthropometric measurements like waist/hip ratio and body fat percentage assessed through bio-electric impedance. Conversely, attempting to elucidate the role of BMI and skeletal body mass in knee osteoarthritis, a study by Sowers et al. [20] found that skeletal muscle mass was highly responsible for the severity of osteoarthritis, compared to fat mass. Nevertheless, King et al. [11] concluded that "precise" evaluation of lean mass did not offer significant advantages over BMI in assessing the risk of radiographic knee osteoarthritis [11].

Holliday et al. (2011) [21] conducted a case-control study related to lifetime BMI and other anthropometric measures of obesity and risk of knee or hip osteoarthritis. In this study, being overweight was deemed a significant risk factor for the majority of patients. Furthermore, rapidly progressive osteoarthritis was commonly observed in the knees, hips, or shoulders but to a lesser extent, in the hand. The same authors concluded that the waist/hip ratio did not correlate with osteoarthritis independently of BMI, except in the female population. On the other hand, waist circumference was found to be associated with a significant risk of lower limb osteoarthritis.

Despite these differences, there are intriguing similarities between the two conditions, both being more prevalent among older adults and potentially occurring prior to certain pain medications [22]. Individuals developing rapidly progressive osteoarthritis show early evidence of cartilage degradation, loss, and signs of inflammation [23]. Additionally, 12% of patients developing AKOA exhibit wear or subchondral fractures [24], while 33% developing accelerated hand osteoarthritis present central and marginal erosion [25]. As rapidly progressive osteoarthritis is accompanied by severe bone destruction, which is not frequently observed in accelerated forms, the exploration of bone morphology related to each type of osteoarthritis might be of crucial importance, providing valuable insights.

On the other hand, there is a growing interest in the role of sedentary behavior as a risk factor for poor health and prevalent diseases, as time spent in a sedentary state is associated with adverse health outcomes, including risks of metabolic disorders [26] such as diabetes mellitus, hypertension, dyslipidemia, diabetes being associated with cardiovascular diseases [27], or musculoskeletal disorders (such as arthritis and osteoporosis). In the National Health and Nutrition Examination Survey (NHANES) sample-based studies, sedentary time correlated with objectively measured BMI and waist circumference, as well as self-reported functional limitations, independently of sedentary time [25].

On the other hand, relieving pain and improving physical condition in patients with osteoarthritis by maintaining a balance between stability and mobility is a crucial aspect in the post-surgery stage. Pharmacological treatment options for osteoarthritis include various types of analgesics. NSAIDs are the most consumed drugs either by prescription or over-the-counter, because of their wide availability and large variety of clinical applications in terms of anti‐inflammatory, analgesic, antipyretic, or antithrombotic effect, being useful as adjuvant therapy for the symptomatic management of the diseases, reducing inflammation and pain [26].

The recommendation of the European Society for Clinical and Economic Aspects of Osteoporosis and Osteoarthritis (ESCEO) is to administrate oral NSAIDs for the management of knee osteoarthritis (OA) in step 2, if step 1 treatment does not show efficacy, or if the patients present moderate-severe pain [27]. The risk of diabetes associated with NSAIDs was not demonstrated, although there are some studies suggesting the association between tramadol and hypoglycemia in diabetic asians [28], while other study reported NSAIDs associated with an increased risk of first-time heart failure hospitalization in patients with T2DM [29]. In another study, the postoperative complications, affecting about 18.23% of patients, mainly involved restricted range of motion (ROM < 90°) and patellar clunk syndrome [30]. Inadequate healing, leading to excessive scar tissue or joint adhesions, may require further surgery. Prosthesis misalignment can restrict joint movement, often requiring surgical revision [30]. The study included different types of postoperative complications such as surgical site infection, metabolic complications, periprosthetic joint infection, and respiratory complications. Significant differences were observed both based on the use of NSAIDs and on metabolic preconditions in the case of surgical site infection.

In the present study, a higher prevalence of diabetes was observed in overweight individuals, particularly those using NSAIDs, as compared to those not using NSAIDs (N-NSAIDs). The level of physical activity shows insignificant differences between N-NSAIDs and NSAIDs in individuals with diabetes. However, among those with metabolic preconditions, there are notable distinctions. Specifically, there is a significant decrease in physical activity in individuals with N-NSAIDs and a significant increase in those using NSAIDs. This phenomenon may be attributed to the short-term benefits of NSAIDs in postoperative mobility. The prevalence of trauma within a three-month period is notably lower with N-NSAIDs, indicating that the long-term administration of anti-inflammatories may offer more benefits. However, it is necessary to support a multimodal approach in order to reduce the side effects of NSAIDs. The association between NSAIDs in the first seven postoperative days, associated with the reduction of collagen secretion, correlated positively, and directly proportional to the incidence of diabetes. Thus diabetes, in any case, associated with delayed healing, if associated with NSAIDs, can affect the recovery period more, implicitly, and the possibility of postoperative complications.

In our study, the majority of patients are sedentary, which may explain not only lower bone mass and obesity but also possible complications of the prosthesis. Thus, we can consider sedentary a significant risk, not only for TKA but also for general health.

Because the study period also included part of the pandemic, when the hospitalization period was significantly reduced, medical recovery may be affected by this aspect. This may represent a limitation of the study. Another limitation pertains to the duration of the study, in which significant complications may still emerge. We intend to conduct a longer-term and more complex study in the future to verify and compare the validity of the results.

## Conclusions

Obesity emerges as a major risk factor for osteoarthritis, emphasizing the importance of managing body weight, especially in individuals with metabolic preconditions who were found to be overweight. Nearly 69% of patients were overweight, while only 31% had a weight considered normal, underscoring the prevalence of weight-related issues, particularly in those without diabetes and without metabolic preconditions. Our study highlights the significant impact of obesity, particularly in individuals with metabolic preconditions, on the risk of osteoarthritis. Differences in complication rates post-operation, notably in those receiving NSAIDs within the first week, suggest potential protective effects associated with certain medication regimens. The prevalence of surgical site infections was notably higher among individuals with metabolic preconditions, emphasizing the importance of considering metabolic status in postoperative care planning. Managing body weight, especially in overweight individuals with metabolic preconditions, is crucial for reducing complications following TKA. Promoting an active lifestyle is also essential for improving arthroplasty outcomes, particularly in individuals without metabolic preconditions.
